# Assessment of alternative genotyping strategies to maximize imputation accuracy at minimal cost

**DOI:** 10.1186/1297-9686-44-25

**Published:** 2012-07-31

**Authors:** Yijian Huang, John M Hickey, Matthew A Cleveland, Christian Maltecca

**Affiliations:** 1 Animal Science Department, North Carolina State University, Campus, Box 7621, Raleigh, NC, 27695, USA; 2 School of Environmental and Rural Science, University of New England, Armidale, Australia; 3 Genus plc., 100 Bluegrass Commons Blvd., Suite 2200, Hendersonville, TN, 37075, USA

## Abstract

**Background:**

Commercial breeding programs seek to maximise the rate of genetic gain while minimizing the costs of attaining that gain. Genomic information offers great potential to increase rates of genetic gain but it is expensive to generate. Low-cost genotyping strategies combined with genotype imputation offer dramatically reduced costs. However, both the costs and accuracy of imputation of these strategies are highly sensitive to several factors. The objective of this paper was to explore the cost and imputation accuracy of several alternative genotyping strategies in pedigreed populations.

**Methods:**

Pedigree and genotype data from a commercial pig population were used. Several alternative genotyping strategies were explored. The strategies differed in the density of genotypes used for the ancestors and the individuals to be imputed. Parents, grandparents, and other relatives that were not descendants, were genotyped at high-density, low-density, or extremely low-density, and associated costs and imputation accuracies were evaluated.

**Results:**

Imputation accuracy and cost were influenced by the alternative genotyping strategies. Given the mating ratios and the numbers of offspring produced by males and females, an optimized low-cost genotyping strategy for a commercial pig population could involve genotyping male parents at high-density, female parents at low-density (e.g. 3000 SNP), and selection candidates at very low-density (384 SNP).

**Conclusions:**

Among the selection candidates, 95.5 % and 93.5 % of the genotype variation contained in the high-density SNP panels were recovered using a genotyping strategy that costs respectively, $24.74 and $20.58 per candidate.

## Background

Successful breeding programs based on genomic information rely on large numbers of animals that are both phenotyped and genotyped at high-density [1,2]. Imputation of high-density genotypes for large numbers of phenotyped animals has been shown to be effective in generating large datasets at lower cost (e.g. [3-5]). Genotyping strategies for imputation generally involve genotyping some individuals in a pedigree at high-density, others at low-density, and in some cases not genotyping other individuals at all. Imputation of genotypes involves two steps. First, the haplotypes carried by the high-density genotyped individuals must be resolved. Then low-density genotypes are used in conjunction with pedigree, familial linkage, and linkage disequilibrium (LD) information to determine the combinations of haplotypes that are carried by animals that are not genotyped or that are genotyped at low-density. Several imputation algorithms have been developed (e.g. *fastPHASE*[6]; *Beagle*[7]; *Phasebook*[8]; *Findhap*[3]; *AlphaImpute*[9]) that vary in accuracy and speed. *AlphaImpute* is sufficiently accurate to permit the use of extremely low-density (e.g. 384 single nucleotide polymorphisms (SNP) across the genome) genotype panels for imputation.

The accuracy of imputation is influenced by several factors, including the number of markers on the low-density genotyping panel, the number of individuals that are genotyped at high-density, the local LD between each low-density genotype and its surrounding high-density genotypes and the number of high-density genotyped relatives of the individuals to be imputed [9-11].

In pedigreed populations, the two major determinants of imputation accuracy are the high-density genotyping status of immediate ancestors and the density of the panel used to genotype the individuals whose genotypes need to be reconstructed [9]. Several alternatives exist to address both these factors. A conservative strategy is to genotype the eight great-grandparents, the four grandparents and the two parents at high-density. This will probably ensure that the phase of the parents is resolved for almost all markers, therefore reducing the task of imputation to the choice of the gamete passed to the offspring and the modelling of recombination events. Furthermore, increasing the density of the low-density genotyping panel reduces the length of the regions for which recombination has to be modelled, resulting in higher imputation accuracy. However, such a conservative strategy can be very costly, especially because in most commercial breeding programs, individual female parents make a relatively small genetic contribution to the next generation. Alternative genotyping strategies can be far less expensive. For example, only male ancestors could be genotyped at high-density and female ancestors at low- or intermediate- density or not be genotyped at all. However, these cheaper alternatives may lead to a sizeable reduction in imputation accuracy.

The objective of this research was to compare the effectiveness of imputation accuracy and the potential cost of alternative genotyping strategies for a commercial breeding program. Specifically, we investigated the imputation accuracy stemming from different sets of ancestors genotyped at high- and low-density, and the interaction between these genotyping strategies and the marker density on imputation candidates. Finally, based on accuracy of imputation of several schemes, the costs of the more relevant of these alternatives were estimated.

## Methods

### Data

To evaluate the accuracy of imputation for various genotyping strategies, data on a set of 98 testing individuals were extracted from a commercial pig-breeding program. These individuals did not have any descendants (i.e. they represented young selection candidates). For each testing individual, both parents and all four grandparents were genotyped at high-density using the Illumina PorcineSNP60 Beadchip. In addition, data on another 2436 genotyped individuals were available. The relationship of individuals from this group (if any) with the testing individuals occurred only through their parents. Genotyped individuals were from a single PIC (a Genus plc. company) nucleus pig line born since 2000, and thus all individuals were moderately to highly related. In this line, individuals were selected for genotyping to target a specific trait in genomic evaluation or were added to fill-in missing herd sires to calculate genomic breeding values. The original selection avoided sampling multiple members of full-sib families. In total, 2779 animals, genotyped at high-density using the Illumina PorcineSNP60 Beadchip, were available. A pedigree of 6473 individuals, consisting of two generations of pedigree for each genotyped animal, was extracted.

Genotypes on a total of 5396 SNP from chromosome 1 with known genome locations were used for analysis after routine editing of the genotype data, which included filtering for extreme minor allele frequency (MAF < 0.001), extreme deviation from Hardy-Weinberg equilibrium (Pearson's Chi-squared test statistic > 300), and proportion of missing genotypes by SNP (> 10 %). Three *in-silico* low-density panels were constructed, with densities equivalent to 6065 (**L6k**), 3022 (**L3k**), and 384 (**L384**) SNP across the entire genome. To select SNP for these panels, 600, 299, and 37 non-overlapping sliding windows of roughly the same size were generated on chromosome 1 for L6k, L3k and L384, respectively. In each sliding window, the SNP with the highest MAF was selected to enter the low-density panel. Summary statistics and assumed costs for each of the low-density panels are given in Table 1. Although only chromosome 1 was analyzed, the results are expected to hold for all chromosomes as in routine genotype imputation work carried out in commercial pig (Matthew Cleveland, unpublished results) and poultry (Andreas Kranis, unpublished results) populations. These studies have employed genotyping strategies and genotype imputation algorithms similar to those used here and very little variation in genotype imputation accuracy has been observed between chromosomes.

**Table 1:** Description of SNP panels for chromosome 1

**SNP panel code**	**SNP panel design**^**1**^	**Number of SNP on chromosome 1**	**Equivalent density across the genome**	**Average spacing (kb) ± SD**	**Cost per genotyped animal**
**H**	High density	5 936	60 000	77. 30 ± 65.22	$120
**L6k**	89.9 % SNP masked	600	6 065	458. 76 ± 187.79	$48
**L3k**	95.0 % SNP masked	299	3 022	913. 96 ± 402.26	$35
**L384**	99.4 % SNP masked	37	384	7359.28 ± 3403.54	$20

^1^A reduced SNP panel with *m* SNP was designed as selecting the highest MAF SNP in each of *m* non-overlapping sliding windows where *m* has a value of 600, 299 and 37 for reduced panel L6k, L3k and L384, respectively; these sliding windows were evenly spaced windows according to their map distances.

### Alternative genotyping strategies

The genotyped pigs were split into four groups, consisting of the 98 testing individuals, their parents, their grandparents, and the remaining high-density genotyped individuals. As a result of the general population structure, in the parental group, nine sires were also grandsires and nine dams were also granddams. When only one group of animals was used, the overlapping individuals were removed from imputation. The numbers of individuals in each group are given in Table 2.

**Table 2:** Accuracy of imputation for twelve genotyping scenarios

**Scenario**	^**1**^**Genotyping strategy**						^**2**^**Imputation accuracy: R-squared**		
	**Other**	**Grandparents**		**Parents**		**Testing individuals**			
		**MGS + PGS**	**MGD + PGD**	**Sire**	**Dam**				
	**n = 2436**	**n = 63**	**n = 86**	**n = 41**	**n = 73**	**n = 98**	**L6k**	**L3k**	**L384**
**s1**	H	H	H	H	H	L	.996	.990	.967
**s2**	H	H	H	H	L	L	.991	.990	.952
**s3**	H	H	H	L	L	L	.989	.984	.941
**s4**	H	H	L	H	L	L	.991	.985	.935
**s5**	H	H	0	H	0	L	.981	.968	.888
**s6**	H	H	H	0	0	L	.984	.974	.910
**s7**	0	0	0	H	H	L	.958	.937	.870
**s8**	0	0	0	H	L	L	.841	.808	.728
**s9**	0	0	0	H	0	L	.850	.794	.719
**s10**	0	H	H	H	0	L	.988	.977	.910
**s11**	H	L	L	L	L	L	.975	.964	.888
**s12**	H	0	0	0	0	L	.953	.931	.817

^1^Animals were split into groups (ordered by generation) of testing individuals, their parents, and their grandparents; grandparents were further divided into two groups: MGS + PGS which included maternal grandsire and paternal grandsire, and MGD + PGD which included maternal granddam and paternal granddam; the remaining individuals were placed in the “Other” category; groups of animals were either genotyped at high-density (H), low-density (L) or not genotyped (0); ^2^Imputation accuracy (R-squared) for scenarios using SNP panels L6k, L3k and L384 on animals genotyped at low density.

To explore the importance of the high- and low-density genotyping status of immediate ancestors of the testing individuals, twelve genotyping strategies were investigated (Table 2). These included genotyping all ancestors of the testing individuals at high-density, genotyping the male ancestors at high-density and the female ancestors at low-density, and only genotyping the remaining individuals at high-density. Other intermediate strategies that involved genotyping some ancestors (e.g. female ancestors at low-density) were also investigated. These twelve scenarios were each tested for all low-density panels created.

In order to investigate the influence of having high-density genotypes on individuals who are neither parents nor grandparents of the testing individuals, three of the twelve scenarios were further expanded (Table 3). These additional scenarios were created by removing (a) none, (b) a random 50 %, or (c) a random 75 % of the high-density genotyped individuals in the group that were not parents or grandparents of the testing individuals.

**Table 3:** Accuracy of imputation for genotyping scenarios when removing subsets of individuals from the “Other” category

**Scenario**	**Genotyping strategy**^**1**^						^**2**^**Imputation accuracy: R-squared**		
	^**3**^**Other**	**Grandparents**		**Parents**		**Testing individuals**			
		**MGS + PGS**	**MGD + PGD**	**Sire**	**Dam**				
	**n2436**	**n = 63**	**n = 86**	**n = 41**	**n = 73**	**n = 98**	**L6k**	**L3k**	**L384**
**s4_100%**	100 % H	H	L	H	L	L	.991	.985	.935
**s4_50%**	50 % H	H	L	H	L	L	.991	.984	.927
**s4_25%**	25 % H	H	L	H	L	L	.988	.981	.915
**s5_100%**	100 % H	H	0	H	0	L	.981	.968	.888
**s5_50%**	50 % H	H	0	H	0	L	.981	.968	.877
**s5_25%**	25 % H	H	0	H	0	L	.979	.966	.871
**s12_100%**	100 % H	0	0	0	0	L	.953	.931	.817
**s12_50%**	50 % H	0	0	0	0	L	.941	.914	.778
**s12_25%**	25 % H	0	0	0	0	L	.917	.879	.759

^1^Animals were split into groups (ordered by generation) of testing individuals, their parents, and their grandparents; grandparents were further divided into two groups: MGS + PGS which included maternal grandsire and paternal grandsire, and MGD + PGD which included maternal granddam and paternal granddam; the remaining individuals were placed in the “Other” category; groups of animals were either genotyped at high density (H), low density (L) or not genotyped (0); ^2^Imputation accuracy (R-squared) for scenarios using SNP panels L6k, L3k and L384 on animals genotyped at low density; ^3^100 % H means that all of the individuals in the “Other” category are genotyped at high density, 50 % H means that only a random 50 % of the individuals in the “Other” category are genotyped at high density, 25 % H means that only a random 25 % of the individuals in the “Other” category are genotyped at high density.

Considering a general livestock population structure where male parents produce a disproportionately large number of progeny compared to females, a number of scenarios emerged from the initial explorations that appeared more suitable for application in the commercial animal-breeding sector. The most suitable scenarios included genotyping selection candidates at very low-density, genotyping male parents at high-density and re-genotyping female parents at high- or medium-density (e.g. from L384 to L6k panels) once they have become parents. Therefore, in this part of the analysis, the use of different low-density panels for female ancestors was explored (Table 4).

**Table 4:** Accuracy and costs of imputation for different genotyping scenarios

**Scenario**	**Genotyping strategy^1^**						**Cost: $**	**Imputation accuracy: R-squared**
	**Other**	**Grandparents**			**Parents**		**Testing individuals**		
		**MGS + PGS**	**MGD + PGD**	**Sire**	**Dam**				
**CostA**	H	H	0	H	0	L384	−^2^	.888
	H	H	L384	H	L384	L384	20.58	.935
	H	H	L3k	H	L3k	L384	24.74	.955
	H	H	L6k	H	L6k	L384	26.28	.956
	H	H	H	H	H	L384	34.84	.967
	H	H	0	H	0	L3k	−^2^	.968
	H	H	L384	H	L384	L3k	−^2^	.980
	H	H	L3k	H	L3k	L3k	35.58	.985
	H	H	L6k	H	L6k	L3k	41.28	.988
	H	H	H	H	H	L3k	49.84	.990
	H	H	0	H	0	L6k	−^2^	.981
	H	H	L384	H	L384	L6k	−^2^	.987
	H	H	L3k	H	L3k	L6k	−^2^	.991
	H	H	L6k	H	L6k	L6k	48.58	.991
	H	H	H	H	H	L6k	62.84	.996
	H	H	H	H	H	H	120.00	1.000

^1^Animals were split into groups (ordered by generation) of testing individuals, their parents, and their grandparents; grandparents were further divided into two groups: MGS + PGS which included maternal grandsire and paternal grandsire, and MGD + PGD which included maternal granddam and paternal granddam; the remaining individuals were placed in the “Other” category; gGroups of animals were genotyped with high density (H), L384, L3k, L6k panels or not genotyped (0); ^2^Represents a scenario that would require the dam of the candidate to be re-genotyped at a lower-density than it would have been originally genotyped when it was itself a selection candidate and this would not occur in practice.

The costs of the alternative genotyping strategies were calculated assuming prices of $120, $48, $35, and $20, for the high-density, L6k, L3k and L384 panels, respectively. Costs were calculated on the basis of an ongoing breeding program, so that for any given generation new genotyping was only relevant for selection candidates and sometimes their parents. For the parents, genotyping, if required, entailed obtaining higher density information compared to that obtained for the same individuals as selection candidates. As a result, the costs of genotyping other ancestors (e.g. grandparents) would be already covered and included when these individuals were themselves parents or candidates. Costs were calculated on a per individual candidate basis, assuming 100 000 selection candidates, from 480 sires and 11 884 dams. These figures do not necessarily reflect those of different commercial breeding programs. Thus, an EXCEL worksheet is provided in which the costs and ratios can be changed to reflect other situations that may exist in practice [see Additional file 1].

### Imputation of genotypes

Imputation was carried out using the software package *AlphaImpute* (version 1.0) [9], which combines simple phasing rules, long-range phasing, haplotype libraries, segregation analysis, and recombination modelling, to impute genotypes for all loci on the highest-density panel of all animals in a pedigree. The genotypes imputed by *AlphaImpute* take the form of the sum of either fully imputed alleles or allele probabilities. Allele probabilities are used when alleles cannot be fully called as integers due to incomplete information (i.e. close to a recombination location or for some markers of individuals that are distantly related to individuals genotyped at high-density).

### Measurement of performance

Accuracy of imputation was measured as the squared correlation (R-squared) between true and imputed genotypes. The R-squared was chosen because it relates to the amount of variation that the imputed genotypes explain in the masked high-density genotypes.

## Results

The average distances in megabases (Mb) between adjacent SNP that are informative for the imputation of paternal and maternal alleles and the percentage of the genome surrounded by informative SNP for each of the four SNP genotyping panels are presented in Table 5. As the density of the genotyping panel decreased, the proportion of the genome surrounded by informative SNP for the paternal and maternal alleles decreased. For the L384 panel, only 88.8 % (83.4 %) of the genome was surrounded by SNP that were informative for the paternal (maternal) gamete and differences between animals were large. The L6k and L3k panels showed a significantly larger proportion of the genome surrounded by informative SNP and lower sampling variance between individuals.

**Table 5:** Summary of informative SNP

	**Percentage of the genome surrounded by informative SNP**^**1**^**± SD**			**Average distance in Mb between adjacent informative SNP ± SD**		
	**Paternal**	**Maternal**	**Average**^**2**^	**Paternal**	**Maternal**	**Average**^**2**^
**H**	99.29 ± 0.81	98.96 ± 1.14	99.12 ± 1.00	0.22 ± 1.08	0.25 ± 1.25	0.23 ± 1.16
**L6k**	98.47 ± 1.01	98.02 ± 1.20	98.24 ± 1.13	1.31 ± 2.90	1.43 ± 3.31	1.37 ± 3.10
**L3k**	97.67 ± 1.70	96.95 ± 1.94	97.31 ± 1.86	2.46 ± 4.31	2.63 ± 4.79	2.54 ± 4.54
**L384**	88.75 ± 8.16	83.41 ± 9.69	86.08 ± 9.33	18.36 ± 18.19	18.99 ± 17.70	18.66 ± 17.96

^1^ Informative SNP: SNP having paternal and maternal alleles inheritance established; genome surrounded by informative SNP means that on one chromosome, the largest section of genome that has informative SNP on both sides.

^2^The average of paternal and maternal.

Accuracy of imputation for the different scenarios is reported in Tables 2, 3, and 4. In all the scenarios, the accuracy was moderate to high and, as expected, it was affected by both the high-density genotyping status of the immediate ancestors and by the density of the panel used to genotype both the testing individuals and their immediate ancestors. Across the twelve basic scenarios (Table 2), the R-squared ranged from 0.996 for s1 (the scenario in which all parents, grandparents, and the remaining individuals were genotyped at high-density and the testing individuals were genotyped with the low-density L6k panel) to 0.719 for s9 (the scenario in which only sires were genotyped and the testing individuals were genotyped with the very low-density L384 panel).

All twelve scenarios showed relatively small differences between the L6k and the L3k panels (e.g. 0.996 for L6k and 0.990 for L3k for scenario s1; 0.953 for L6k and 0.931 for L3k for scenario s12). However, the L384 panel was noticeably less accurate than the L3k or L6k panels (e.g. 0.990 for L3k and 0.967 for L384 for s1; 0.931 for L3k and 0.817 for L384 for s12). The overall accuracy decreased and the differences in accuracy among the panels increased as the amount of high-density genotyping in the ancestral relatives decreased. Once the parents of the testing individuals were genotyped at high-density, there was little benefit in having other ancestral relatives genotyped (i.e. scenario s7 was almost as accurate as scenario s1, except for the very low-density scenario). In scenario s6 (i.e. ancestral relatives but not the parents are genotyped at high-density), low accuracies were again obtained when the L384 panel was used for the testing individuals. Genotyping the parents with the same low-density panel as the candidates (scenario s3) recovered some of this loss. In comparison to scenario s6 (i.e. no genotyping of parents), which had accuracies of 0.984, 0.974, and 0.910 for the L6k, L3k, and L384 panels respectively, scenario s3 (i.e. parents are genotyped at low-density) had accuracies of 0.989, 0.984, and 0.941. Extending the low-density genotyping to the grandparents (scenario s11) resulted in a notable loss in accuracy compared to limiting the use of the low-density panel to the parents only (scenario s3). When compared to using high-density genotyping on both male and female ancestors (scenario s1), genotyping the female ancestors at low-density (i.e. the dam and granddams) and genotyping the male ancestors at high-density (i.e. the sire and grandsires) (scenario s4) resulted in small losses in imputation accuracy, even when using the L384 panel on the testing individuals. When the grandparents and other ancestors were not genotyped, a considerable loss was observed when the dam was not genotyped at high-density, especially when the L384 panel was used on testing individuals, as shown by the comparison of scenarios s7, s8, and s9.

The effect of having high-density genotypes on ancestral relatives that are not parents or grandparents on the accuracy of imputation is shown in Table 3. For scenarios s4 (i.e. sire and grandsires genotyped at high-density and dam and granddams at low-density) and s5 (i.e. sire and grandsires genotyped at high-density and dam and granddams not genotyped), no effect was observed when all the other 2436 individuals in the dataset were used for imputation, as opposed to using a random subset of 50 % or 25 % of them. For scenario s12 (i.e. no genotyping of parents or grandparents), decreasing the “other” group from 100 % to 50 % and 25 % produced only a small effect when the low-density L6k and L3k panels were used to genotype the testing individuals but a large effect when the low-density L384 panel was employed.

This initial analysis suggested that a practical genotyping strategy for a commercial breeding program could consider genotyping male parents at high-density and female parents at high- or low-density. Candidates to selection could themselves be genotyped with one of the low-density panels. The accuracy of imputation and the costs per individual of each of these scenarios are shown in Table 4. When the testing individuals were genotyped with the L6k panel, there was little difference in accuracy of imputation between genotyping dams and granddams with the high-density panel, the low-density L6k, L3k, L384 panels or not genotyping them at all (0.981 - 0.996). Small differences in accuracy were observed between strategies for genotyping dams and granddames when testing individuals were genotyped with the L3k panel, while larger differences were observed with the L384 panel. Not genotyping the dams and granddams and genotyping the testing individuals with panel L384 gave an accuracy of 0.888, while accuracies of 0.935, 0.955 and 0.956 were obtained by adding L384, L3k and L6k genotypes for the dam and granddams, respectively. By comparison, an accuracy of 0.967 was achieved when the dams were genotyped at high-density. The costs of these scenarios ranged from $20.58 to $34.84 per individual and were substantially lower than the cost of genotyping every candidate at high-density ($120). Three factors influenced the genotyping costs of a scenario: the price of the low-density panel used to genotype candidates, the number of offspring produced by a female parent coupled with the cost of genotyping this female, and the number of offspring produced by the male parent coupled with the cost of genotyping the male parent at high-density. Of these factors, the cost associated with the male parent was the least important because of the large numbers of offspring produced by sires. In general, costs were sensitive to all of these parameters and an Excel spreadsheet is supplied in Additional file 1 [see Additional file 1] that can be used to evaluate alternative prices of the different genotyping panels and alternate reproductive ratios of males and females.

## Discussion

For the purposes of pedigree-based genotype imputation, several strategies involving genotyping male and female ancestors of candidates for selection at various high- or low-densities and the candidates themselves at various low-densities were evaluated. The results demonstrate that most of the information contained within the high-density genotyping panels can be recovered using low-cost genotyping strategies such as genotyping the candidates for selection at a very low-density (i.e. a 384 SNP panel), the female parents at a very to moderately low-density (i.e. a 384 or 3000 SNP panel), and genotyping male parents at a high-density. Furthermore, the costs of initiating such a genotyping strategy in a new line of animals would be low because genotyping large numbers of individuals at high-density does not appear to be required once the male and female parents (or the maternal-grandsires) of the generation for which the strategy is implemented are genotyped at high-density.

Imputation of genotypes involves two steps: (1) determining the phase of high-density haplotypes and (2) determining which combination of these haplotypes are carried by an individual genotyped at low-density and modelling any recombination that occurs during the meiosis that created this individual. These two steps have different impacts on the accuracy and costs of imputation and the different genotyping strategies tested in this study illustrate this.

To obtain accurate phasing of the high-density genotypes of key ancestors, it is necessary to genotype other individuals at high-density. *AlphaImpute* uses a phasing algorithm (*AlphaPhase*[12] – long-range phasing and haplotype library imputation) that does not require restrictive high-density genotyping strategies (e.g. multiple generations of ancestors genotyped at high-density). Previously, it has been shown that for *AlphaPhase* to give accurate phasing results, it requires at least 1000 high-density genotyped individuals [12]. However, the results of this study show that, within the *AlphaImpute* framework, highly accurate imputation can be obtained once the parents, or the sire and maternal-grandsire of the selection candidates are genotyped at high-density, without the need for a large pool of individuals genotyped at high-density. There are two reasons for this. First, *AlphaImpute* incorporates a number of phasing error detection steps that were not included in *AlphaPhase*. Second, *AlphaImpute* implements some simple pedigree-based phasing rules that interact with the other phasing procedures to eliminate much of the phasing errors. The ability to accurately impute genotypes from such a small training population considerably reduces the costs of initialising a genomic selection program based on imputation in a new line that has not been previously genotyped at high-density.

Determining the high-density haplotypes carried by an individual genotyped at low-density and modelling recombination were relatively accurate once the parents were genotyped at high-density. For more complex scenarios (i.e. female ancestors not genotyped at high-density), having some level of genotyping on the female ancestors increased the accuracy of the imputation as shown in Table 4. Several recombination events occur during meiosis and accurate imputation requires identification and modelling of these events. When using low-density SNP panels (e.g. 384 SNP) for imputation, there are relatively few informative SNP (Table 5) and therefore large regions surrounding a recombination event may not have information for the purposes of imputation. With multiple generations of low-density genotyping on one or both sides of the pedigree, the overall proportion of the genome that includes a recombination event between a pair of informative SNP increases. This severely restricts imputation accuracy of genotyping strategies that make use of very low-density SNP panels (e.g. 384 SNP) to genotype parents or grand-parents of selection candidates.

Commercial breeding programs aim at maximising the rate of genetic gain within cost constraints. Genomic information offers great potential for increased rates of gain but the cost of realizing that potential can be high, especially if large numbers of selection candidates need to be genotyped or parents have relatively few offspring and the cost of genotyping them is therefore spread across relatively few individuals, as is the case in pig and poultry breeding programs. The costs of alternative genotyping strategies presented here are specific to the assumptions made in relation to the costs of the different genotyping panels and the numbers of offspring produced by male and female parents. Small changes in these factors can have big impacts on the relative costs of different strategies and this can be explored using the excel spreadsheet provided in Additional file 1 [see Additional file 1]. Ninety-five percent of the genotype variation among the selection candidates contained in the high-density SNP panels could be recovered at a cost of $24.74 per candidate when using a genotyping strategy that involved genotyping male parents at high-density, female parents at low-density (e.g. 3000 SNP), and selection candidates at very low-density (384 SNP), and the mating and offspring per parent ratios described in the additional file (480 sires; 11884 dams and 10000 offspring). However, results will depend on species-specific characteristics. For example, in a hypothetical sheep breeding program scenario in which five males and 250 females are used to produce 300 candidates for selection, the same strategy would cost $51.17 per candidate.

While the results of this study show that most of the information content of full high-density genotyping can be recovered using low-cost genotyping strategies, the effect that this will have on the accuracy and bias of the resulting estimated breeding values is unknown and deserves further study since decisions on investment cannot be made based on costs alone. Furthermore, imputation errors may affect the different components of the estimated breeding values differently. Imputation error or loss of information due to incomplete imputation could impact the accuracy of the estimated Mendelian sampling term only and not the parental average component or it might in turn influence only the accuracy of the dam’s contribution to the estimated breeding value. Under these circumstances, the advantage of genomic over pedigree information for delivering higher rates of gain at reduced levels of inbreeding will be decreased. Furthermore, if imputation accuracy is unevenly distributed across the genome, parts of the genome could potentially be less accurately selected upon and therefore be subject to greater random genetic drift over time.

The proportion of the genome that was covered by low-density SNP that were informative for imputation decreased when going from high- to low-density scenarios. This decrease was moderate for L6k and L3k panels, but approximately 13 % of the genome was not covered in the L384 scenarios. This results in approximately 6 % of the genome at each end of a chromosome not being informative for imputation, regardless of the imputation method employed. Thus, when designing extremely low-density marker panels (e.g. L384) allocating more markers at the ends of the chromosomes could be advantageous.

It could be that the high imputation accuracies observed in this study are partially explained by the high level of relationships among individuals of the population analysed, particularly for scenarios where immediate parents were not genotyped at high-density. In this case, imputation requires that the haplotypes of the individuals to be imputed are (at least partially) represented in the haplotype libraries. However, high relationships between individuals in the population are likely not needed for accurate imputation when the parents or grandparents are genotyped at high-density, since good performance of the phasing algorithm does not depend on high levels of relatedness between the high-density individuals, as shown by Hickey et al. (2011), and the imputation does not depend on information from other individuals once the parents or grandparents are genotyped at high-density.

## Conclusions

Commercial breeding programs seek to maximise genetic gain while minimising the costs of attaining that gain. Low-cost genotyping strategies involving genotype imputation offer dramatically reduced costs for the implementation of genomic selection. However, both costs and accuracy of imputation of these strategies are highly sensitive to several factors. Given the mating ratios and numbers of offspring produced by males and females, a low-cost genotyping strategy for a commercial pig population could involve genotyping male parents at high-density, female parents at low-density (e.g. 3000 SNP), and selection candidates at very low-density (384 SNP). Among the selection candidates, 95.5 % and 93.5 % of the genotype variation contained in the high-density SNP panels were recovered using a genotyping strategy that costs respectively $24.74 and $20.58 per candidate.

## Competing interests

The authors declare that they have no competing interests.

## Authors’ contribution

MAC, JMH, and CM conceived and designed the experiment. YH edited the data and performed the analysis. CM and JMH wrote the first draft of the manuscript. All authors read and approved the final manuscript.

## Supplementary Material

Additional file 1 **Accuracy_Cost_Eval.** The EXCEL spreadsheet provides information on the overall cost-accuracy of different genotyping imputation strategies. It allows varying the number of individual genotyped, the density of genotyping, and the cost per individual genotyped. Click here for file
